# Association of antihypertensive drugs with COVID-19 outcomes: a drug-target Mendelian randomization study

**DOI:** 10.3389/fphar.2023.1224737

**Published:** 2023-12-05

**Authors:** Kun Zhang, Hengxing Gao, Mingwei Chen

**Affiliations:** Department of Respiratory and Critical Care Medicine, The First Affiliated Hospital of Xi’an Jiaotong University, Xi’an, Shaanxi, China

**Keywords:** COVID-19, ACE, ADRB1, CCB, Mendelian randomization, GWAS

## Abstract

**Background:** Observational investigations have provided conflicting results regarding the effect of antihypertensive drugs on the risk of COVID-19 outcomes. We intended to assess the causal effect of antihypertensive drugs on COVID-19 outcomes using drug-target Mendelian randomization (MR), mainly including angiotensin-converting enzyme inhibitors (ACEIs), β-blockers (BBs) and calcium channel blockers (CCBs).

**Methods:** We used the genetic variants (minor allele frequency >1%, *r* ^2^ < 0.30) located within 100 k bases of each drug target gene and associated with lower systolic blood pressure (*p* < 5 × 10^−8^) as genetic proxies for antihypertensive drugs. COVID-19 outcomes included COVID-19 susceptibility (122,616 cases and 2,475,240 controls), hospitalization (32,519 cases and 206,2805 controls), and severe illness (13,769 cases and 1,072,442 controls). All studies were conducted on populations of European ancestry. MR estimates were generated using an inverse variance weighted (IVW) model.

**Results:** IVW-MR analysis observed a weak causality between CCBs and COVID-19 susceptibility (OR: 0.993, 95% CI: 0.988–0.999, *p* = 0.012). Sensitivity analysis suggested that this result was robust. No evidence was found for a link between other antihypertensive drugs and COVID-19 outcomes.

**Conclusion:** The present study suggests that CCBs may reduce COVID-19 susceptibility in European populations.

## 1 Introduction

The COVID-19 pandemic has resulted in hundreds of millions of infections and millions of deaths (Center JHCR, 2023). Thus, identifying risk factors is essential to improve the outcome of COVID-19.

Available studies suggest that individuals with comorbidities are at higher risk of infection, hospitalization, and death from COVID-19, including those with hypertension (Leung et al., 2020; Semenzato et al., 2021; Zhou et al., 2021). These results seem to indicate a possible beneficial effect of antihypertensive drugs on COVID-19. Current studies about the effect of antihypertensive drugs in influencing the outcomes of COVID-19 have shown inconsistent or even opposing conclusions. Some studies claim that using ACEIs and angiotensin receptor blockers (ARBs) lowers the likelihood of mortality and hospitalization in COVID-19 patients (Zhang P. et al., 2020; Semenzato et al., 2021). In contrast, other studies concluded that ACEIs and ARBs were unrelated to increased poor outcomes in COVID-19 patients (Fosbøl et al., 2020; Mackey et al., 2020; Reynolds et al., 2020). Even some investigators reasonably suspect that ACEIs and ARBs may increase ACE2 expression and increase viral entry into host cells (Patel and Verma, 2020). The effect of β-blockers (BBs), calcium channel blockers (CCBs), and thiazide diuretics on COVID-19 outcomes is also controversial (Al-Kuraishy et al., 2021; Matli et al., 2022; Wojciechowska et al., 2022). Several studies have found that CCBs may reduce severe illness and mortality in COVID-19 (Crespi and Alcock, 2021; Loas et al., 2022). However, other investigators have found a significantly increased risk of intubation or death in patients taking dihydropyridine CCBs (Mendez et al., 2021). In short, many observational surveys analyzed the relationship between antihypertensive drugs and COVID-19, yet have not reached consistent conclusions. Moreover, these investigations are vulnerable to inverse causality and confounders.

Drug-target Mendelian randomization (MR) analysis simulates genetic variation in pharmacological suppression of drug targets, reflecting the impact of drug use. MR study can minimize confounding bias and avoid inverse causality (Lawlor et al., 2008).

In the present study, based on the latest released genome-wide association study (GWAS) summary data for COVID-19, we used drug-target MR to assess the causal effects of antihypertensive drugs on COVID-19 outcomes.

## 2 Materials and methods

### 2.1 Study design

**FIGURE 1:**
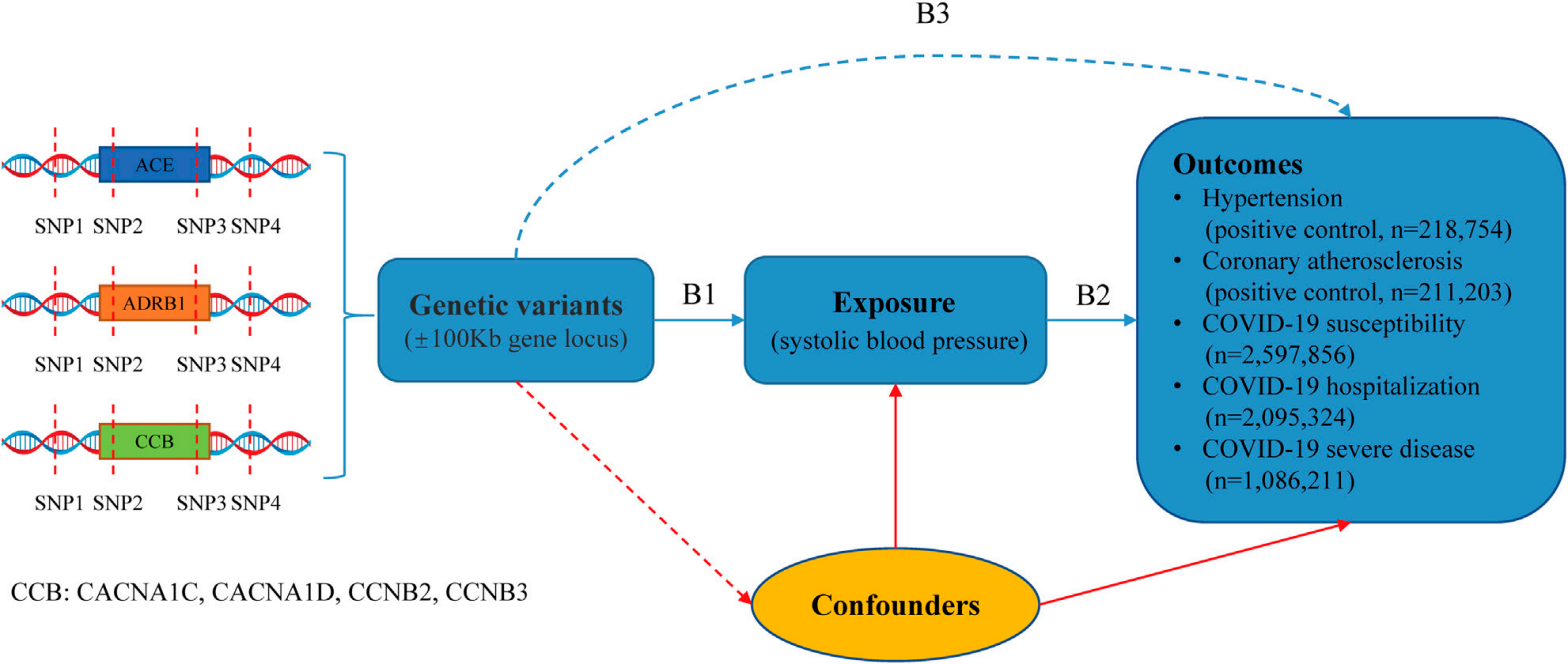
Drug-target Mendelian randomization study design.

The research design is illustrated in Figure 1. We conducted a drug-target MR analysis utilizing publicly available GWAS summary data to assess the impact of antihypertensive drugs on outcomes. The GWAS for systolic blood pressure (SBP) and diastolic blood pressure (DBP) was obtained from the UK Biobank and International Consortium of Blood Pressure (Evangelou et al., 2018). Hypertension and coronary atherosclerosis GWAS data derived from the FinnGen database (https://www.finngen.fi/fi). Moreover, the COVID-19 outcomes were sourced from the COVID-19 Host Genetics Initiative (https://www.covid19hg.org/results/r7/). The institutional review boards corresponding to these studies approved these studies and provided written informed consent for participants. Details of the data sources are provided in Supplementary Table S1. In Figure 1, B1 and B3 are estimates of instrumental variables on exposures (antihypertensive drugs) and outcomes. B2 is the long-term effect of antihypertensive drugs on outcomes, which interests us, and B2 = B3/B1 estimates this association.

### 2.2 Genetic instruments for antihypertensive drugs

To proxy antihypertensive drugs, we utilized single nucleotide polymorphisms (SNPs) situated within 100 k bases of each drug’s target genes (minor allele frequency >1%) and associated with lower SBP from the SBP GWAS (N = 757,601) with *p* < 5 × 10^−8^. The target genes of ACEIs, ARBs, BBs, CCBs, and thiazide diuretics are shown in Table 1. In addition, instrumental SNPs can be in low linkage disequilibrium (*r* ^2^ < 0.30). We also crudely calculated the F-statistic to validate if these SNPs were strong instruments (Burgess et al., 2011). Antihypertensive drugs without compliant genetic instruments will not be included in MR analysis.

**TABLE 1:** Genetic regions and the number of instrumental variables of antihypertensive drug targets.

Exposure drug target	Gene	Gene position (GRCh37/hg19)	N(SNP)
Angiotensin-converting enzyme inhibitors	ACE	Chr17: 61554422–61575734	1
β-blockers	ADRB1	Chr10: 115803625–115806663	12
Calcium channel blockers	CACNA1C	Chr12: 2162153–2807116	2
	CACNA1D	Chr3: 53528638–53847760	10
	CACNA1F	ChrX: 49061523–49089802	0
	CACNA1S	Chr1: 201008640–201081554	0
	CACNA2D1	Chr7: 81575760–82073272	0
	CACNA2D2	Chr3: 50400044–50541675	0
	CACNB1	Chr17: 37329706–37353922	0
	CACNB2	Chr10: 18429353–18832486	43
	CACNB3	Chr12: 49208263–49222724	2
	CACNB4	Chr2: 152689285–152955681	0
	CACNG1	Chr17: 65,040,670–65052913	0
Angiotensin receptor blockers	AGTR1	Chr3: 148415690–148460790	0
Thiazide diuretics	SLC12A3	Chr16: 56899119–56949762	0

### 2.3 Outcomes

COVID-19 GWAS were acquired from COVID-19-hg GWAS meta-analyses round 7 (latest data). Of these, the COVID-19 susceptibility sample included 2,475,240 controls and 122,616 cases. The COVID-19 hospitalization sample consisted of 2,062,805 controls and 32,519 cases, and the COVID-19 severe disease sample consisted of 1,072,442 controls and 13,769 cases. We used hypertension data (162,837 controls and 55,917 cases) and coronary atherosclerosis (187,840 controls and 23,363 cases) as positive controls to test the effectiveness of instrumental SNPs. All genetic associations were based on cohorts of European ancestry. Details of outcomes are provided in Supplementary Table S1.

### 2.4 Statistical analysis

In GWAS studies of SBP, instrumental SNPs were identified in or close to the locations of drug-targeted genes as genetic instruments used to perform drug-target MR. MR estimates of SBP reduction by antihypertensive drugs on COVID-19 outcomes were yielded by the inverse variance weighted (IVW) method (Burgess et al., 2017). When only one variant exists, MR estimates are generated by the Wald ratio. Beta coefficient or odds ratios with 95% confidence intervals are used to present the results.

For the IVW approach, we performed the Cochran'Q test to examine the heterogeneity of the instrumental variables. If heterogeneity existed, we used a random effect IVW model. In MR Egger regression, the intercept term was utilized to identify the pleiotropy. Standardized MR estimates and 95% confidence intervals for this drug represent each 10-mmHg reduction in SBP by drug target genes. When an antihypertensive drug is causally associated with COVID-19 outcomes, explore whether other genetic variants related to SBP are causally linked to that COVID-19 outcome. This will help us to understand whether the drug is independent of its antihypertensive effect. If CCBs have a causal effect on COVID-19 through a “direct effect” (i.e., independent of blood pressure), then we would expect to see significant causality estimates in MR analyses involving SNPs in the CCB genes but not in analyses involving SNPs in ACE, BB, or genes in the rest of the genome. If the effect of SNPs on COVID-19 is mediated through blood pressure, then we would expect to obtain significant causality estimates using SNPs associated with blood pressure in other parts of the genome. Also, we conducted the “leave-one-out” method to assess a robustness analysis of the significant MR estimates. In addition, we utilized the DBP GWAS study (N = 757,601) in this hypertension cohort to validate the results of the MR analyses.

All statistical analyses described above were conducted utilizing the “TwoSampleMR” package (version 0.5.6) in the R (version 4.2.1). *p* < 0.05 was considered significant.

## 3 Results

As shown in Table 1, we obtained 1 and 12 SNPs as genetic proxies for ACEIs and BBs, respectively. For CCBs, we acquired a total of 57 genetic instrumental variables for 11 genes in their targets. While ARBs and thiazide diuretics had no suitable genetic proxy and were excluded from MR analysis. All instrumental SNPs had F-statistics greater than 10 (Supplementary Table S2).

In order to simulate the pharmacological effects of these drugs, we have corresponded all MR effect estimates to a decrease in SBP. First, in the positive control analyses, the ACEI, BB, and CCB genetic instruments all reduced the risk of hypertension and coronary atherosclerosis (Table 2; Supplementary Table S3). The results of heterogeneity, pleiotropy, and the “leave-one-out” results are illustrated in Supplementary Table S4 and Supplementary Figure S1.

**TABLE 2:** Potential effects of ACEIs, BBs and CCBs on COVID-19 outcomes.

Outcome	Drug	N(SNP)	Beta (95% CI)	OR (95% CI)	*P* Value
**Positive control**					
Hypertension	ACEIs	1	−0.126 (−0.189–0.064)	0.881 (0.828–0.938)	7.25E-05
	BBs	12	−0.152 (-0.181–0.123)	0.859 (0.834–0.884)	1.57E-24
	CCBs	56	−0.093 (-0.108–0.079)	0.911 (0.898–0.924)	1.14E-36
Coronary atherosclerosis	ACEIs	1	−0.090 (-0.176–0.004)	0.914 (0.839–0.996)	0.040
	BBs	12	−0.046 (-0.075–0.017)	0.955 (0.928–0.983)	0.002
	CCBs	57	−0.017 (-0.031–0.003)	0.983 (0.969–0.997)	0.016
**COVID-19 outcome**					
COVID-19 susceptibility	ACEIs	1	0.007 (-0.025–0.040)	1.007 (0.975–1.041)	0.659
	BBs	12	−0.001 (0.012–0.01)	0.999 (0.988–1.010)	0.843
	CCBs	57	−0.007 (-0.012–0.001)	0.993 (0.988–0.999)	0.012
COVID-19 hospitalization	ACEIs	1	0.019 (-0.052–0.089)	1.019 (0.949–1.094)	0.603
	BBs	12	0.003 (-0.019–0.026)	1.003 (0.982–1.026)	0.758
	CCBs	57	−0.006 (-0.018–0.005)	0.994 (0.982–1.005)	0.280
COVID-19 severe disease	ACEIs	1	0.049 (-0.053–0.151)	1.050 (0.948–1.163)	0.349
	BBs	12	0.004 (-0.037–0.045)	1.004 (0.964–1.046)	0.854
	CCBs	57	0.000 (-0.018–0.019)	1.000 (0.982–1.020)	0.964

Abbreviations: ACEI: angiotensin-converting enzyme inhibitor, BB: β-blocker; CCB: calcium channel blocker.

MR analysis of COVID-19 showed that neither ACEIs nor BBs showed a significant causal effect on COVID-19 outcomes (Table 2; Supplementary Table S5). In contrast, CCBs showed a beneficial effect on COVID-19 susceptibility (Table 2, OR: 0.993, 95%CI: 0.988–0.999, *p* = 0.012). Moreover, after Bonferroni correction (*p* = 0.05/3), a statistically significant difference remained. The Cochran'Q test and MR-Egger regression indicated no heterogeneity and pleiotropy (Supplementary Table S6). Moreover, the “leave-one-out” analysis further confirmed the result’s robustness (Supplementary Figure S1). We also assessed the effect of instrumental SNPs other than CCB targets on COVID-19 susceptibility, and the results did not show a causal association (Supplementary Table S7). In addition, CCBs did not show a beneficial effect on the risk of COVID-19 hospitalization and serious illness (Table 2). To further validate the main findings of this MR analysis, we validated the causal estimation of antihypertensive drugs and COVID-19 outcomes in the diastolic blood pressure cohort and found that CCBs still causally reduced COVID-19 susceptibility (Supplementary Table S8).

## 4 Discussion

The present MR study analyzed the effect of antihypertensive drugs on COVID-19 outcomes. Our results provide suggestive evidence about the possibility of CCBs in reducing COVID-19 infection risk. In a complementary analysis of SBP and COVID-19 susceptibility, we did not observe a significant link, suggesting that CCB reduces the risk of COVID-19 infection independent of SBP. In addition, we did not obtain evidence of genetic association in the MR analysis of antihypertensive drugs with other COVID-19 outcomes.

**FIGURE 2:**
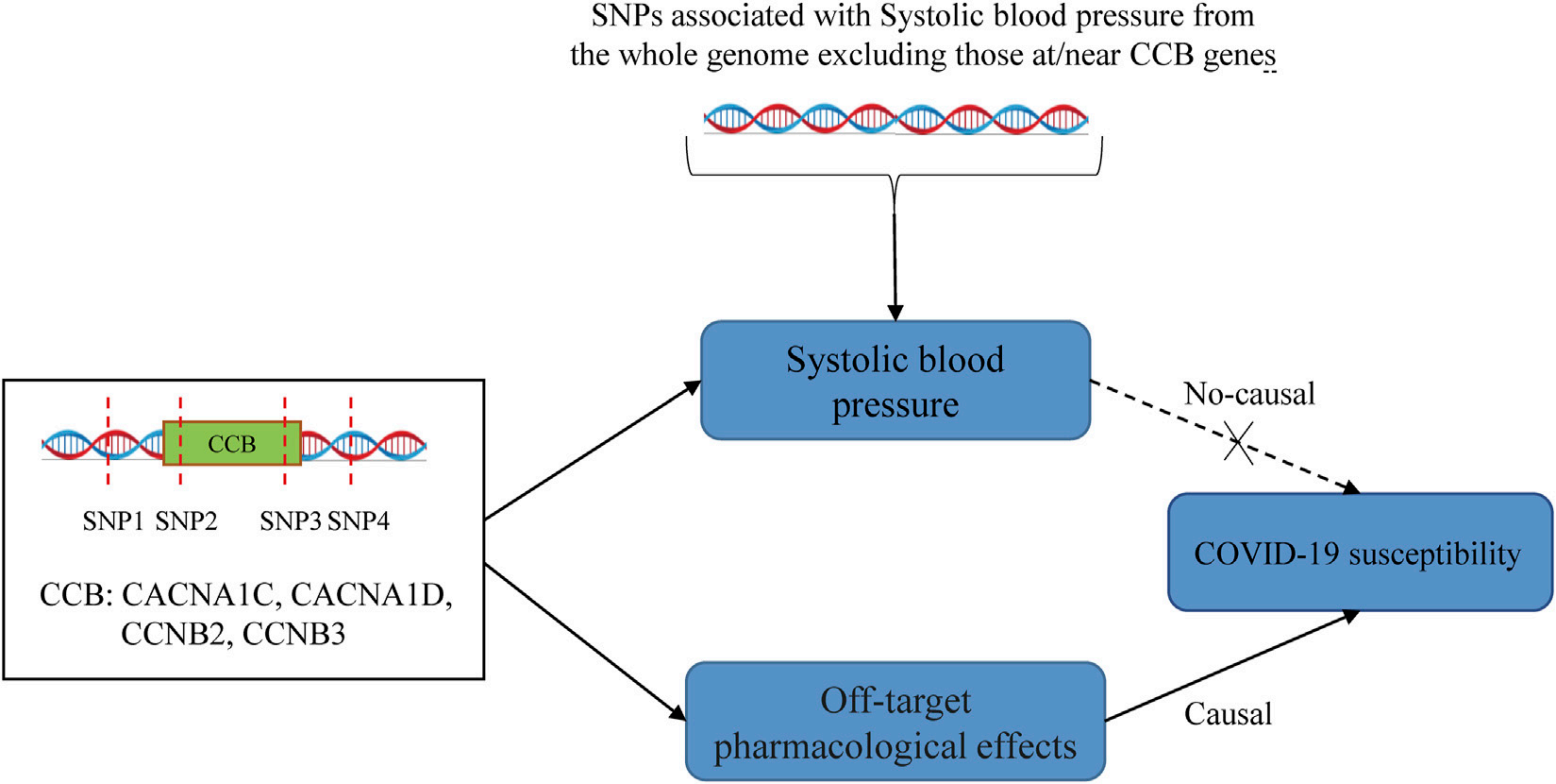
The mechanism of the drug-target Mendelian randomization.

COVID-19 patients with comorbidities have worse outcomes. This has prompted a search for the possibility of improving COVID-19 outcomes in drugs used to treat comorbidities. Thus, drug repurposing has received attention during the COVID-19 pandemic (Gaziano et al., 2021). Antihypertensive drugs may have a role in antivirals based on the pleiotropic effects of antihypertensive drugs, such as anti-inflammatory, immunomodulatory, and calcium metabolism (Crespi and Alcock, 2021; Lim, 2021; Bryniarski et al., 2022). ACEIs and ARBs were the first to attract attention because of the critical role of ACE2 in SARS-CoV2 infection. Our drug-target MR study did not observe a causality between ACEIs and COVID-19 outcomes. This is consistent with an observational study published in NEJM, and two other studies also support our findings (Fosbøl et al., 2020; Mancia et al., 2020; Xu et al., 2021). Although some observational studies concluded that ACEIs reduced hospitalization and mortality in COVID-19 patients (Zhang P. et al., 2020; Semenzato et al., 2021), the confounding bias of these studies could not be avoided and did not allow causal inference, which the MR study could overcome. Some studies have found that BBs prevent fatal complications in COVID-19 patients through anti-inflammatory effects and attenuation of catecholamine release (Al-Kuraishy et al., 2021; Alsagaff and Mulia, 2021). However, other clinical observations found no significant link between BBs and COVID-19 outcomes (Iaccarino et al., 2020; Reynolds et al., 2020), which was confirmed by our MR study. Notably, the relationship between CCBs use and adverse outcomes in COVID-19 patients is controversial (Zhang LK. et al., 2020; Reynolds et al., 2020; Solaimanzadeh, 2020; Mendez et al., 2021), and we did not obtain a significant association between CCBs and COVID-19 hospitalization and severe illness. However, we found that CCBs causally reduced COVID-19 susceptibility. Although the causal association between CCBs and COVID-19 susceptibility was weak (OR: 0.993), we can infer that this association is unlikely to be caused by random factors due to the *p*-value of 0.012 (still significant after Bonferroni correction). Several previous studies have also identified a possible antiviral effect of CCB. (DeWitt and Waksman, 2004; Zhou et al., 2013; Crespi and Alcock, 2021). Moreover, the effect of CCBs in reducing COVID-19 susceptibility was not related to their hypotensive effect (Figure 2). The possible mechanism is that CCBs can reduce viral entry and interfere with viral replication by reducing intracellular and extracellular calcium levels (Crespi and Alcock, 2021).

We evaluated the causal association of antihypertensive drugs with COVID-19 outcomes (latest release) using a drug-target MR approach, which minimizes confounding bias and avoids reverse causality. This is the greatest strength of our study. Of course, our study has some limitations. First, we used GWAS summary data, which limited us to performing subgroup analysis and adjusting for specific covariates. Second, although we conducted several sensitivity analyses to examine the MR hypotheses, pleiotropy could not be totally eliminated. Third, the GWAS data for this study were mainly from European populations, so we should be cautious about expanding the use of these findings.

## 5 Conclusion

In summary, our MR study revealed a weak causality between CCBs and decreased susceptibility to COVID-19. However, further clinical trials and mechanistic studies are needed to validate this finding.

## Data Availability

The original contributions presented in the study are included in the article/Supplementary Material, further inquiries can be directed to the corresponding author.
