# Supplementary material for: Association of antihypertensive drugs with COVID-19 outcomes: a drug-target Mendelian randomization study
Source: Front Pharmacol. 2023 Dec 5;14:1224737. doi: 10.3389/fphar.2023.1224737 (PMC10728283; doi:10.3389/fphar.2023.1224737)
Supplement: Supplementary file 1 [file DataSheet1.docx]

**Supplementary material**

**Association of antihypertensive drugs with COVID-19 outcomes: a drug-target Mendelian randomization study**

Kun Zhang^1^, Hengxing Gao^1^, Mingwei Chen^1, *^

^1^Department of Respiratory and Critical Care Medicine, The First Affiliated Hospital of Xi'an Jiaotong University, Xi'an, 710061, Shaanxi, China.

^*^Correspondence: Mingwei Chen

Department of Respiratory and Critical Care Medicine, The First Affiliated Hospital of Xi'an Jiaotong University, 277#, Yanta West Road, Xi'an, 710061, Shaanxi Province, People's Republic of China.

Tel: 13088988001

Email: chenmw36@163.com

**Index**

**Table S1. Details of phenotype and GWAS.**

**Table S2. Genetic instruments of antihypertensive drugs.**

**Table S3. Results of mendelian randomization analysis related to antihypertensive drugs and positive controls.**

**Table S4. Results of heterogeneity and pleiotropy tests related to antihypertensive drugs and positive controls.**

**Table S5. Results of mendelian randomization analysis related to antihypertensive drugs and COVID-19 outcomes.**

**Table S6. Results of heterogeneity and pleiotropy tests related to antihypertensive drugs and COVID-19 outcomes.**

**Table S7. Mendelian randomization analysis of systolic pressure on COVID-19 susceptibility (other than CCB targets).**

**Table S8. Results of Mendelian randomization analyses validating the association of antihypertensive drugs and COVID-19 outcomes in the diastolic blood pressure cohort.**

**Figure S1. Sensitivity analysis of the results of mendelian randomization.**

**Table S1. Details of phenotype and GWAS.**

| **Phenotype** | **Consortium** | **First Author (Year)** | **Sample Size** | **N Cases** | **N Controls** | **Population** | **MRC-IEU ID:** | **Unit** |
| --- | --- | --- | --- | --- | --- | --- | --- | --- |
| Systolic blood pressure/ diastolic blood pressure | ICBP and UK Biobank | Evangelou,E  (2018) | 757,601 | NA | NA | European | ieu-b-38/ ieu-b-39 | mmHg |
| Hypertension | FinnGen | NA | 218,754 | 55917 | 162837 | European | finn-b-I9_HYPTENS | NA |
| Coronary atherosclerosis | FinnGen | NA | 211,203 | 23363 | 187840 | European | finn-b-I9_CORATHER | NA |
| COVID-19 susceptibility | COVID-19 Host Genetics Initiative | NA | 2,597,856 | 122616 | 2475240 | European | NA | NA |
| COVID-19 hospitalization | COVID-19 Host Genetics Initiative | NA | 2,095,324 | 32519 | 2062805 | European | NA | NA |
| COVID-19 severe disease | COVID-19 Host Genetics Initiative | NA | 1,086,211 | 13769 | 1072442 | European | NA | NA |

MRC-IEU: Medical Research Center-Integrative Epidemiology Center (University of Bristol, UK); UKB: UK Biobank; ICBP: International Consortium of Blood Pressure.

**Table S2. Genetic instruments of antihypertensive drugs.**

| **Drug target** | **SNP** | **chr** | **pos** | **beta** | **se** | **pval** | **samplesize** | **Effect allele** | **Other allele** | **eaf** | **F** |
| --- | --- | --- | --- | --- | --- | --- | --- | --- | --- | --- | --- |
| ACE | rs8077276 | 17 | 61547562 | -0.2949 | 0.0314 | 5.15E-21 | 722573 | A | G | 0.6201 | 88.2044 |
| ADRB1 | rs740746 | 10 | 115792787 | 0.4557 | 0.0342 | 1.42E-40 | 725665 | A | G | 0.7318 | 177.5439 |
| ADRB1 | rs10885531 | 10 | 115814392 | 0.2999 | 0.0302 | 3.63E-23 | 728049 | T | C | 0.5009 | 98.61411 |
| ADRB1 | rs855715 | 10 | 115823524 | -0.4394 | 0.0482 | 7.88E-20 | 688410 | T | G | 0.1202 | 83.10478 |
| ADRB1 | rs143854972 | 10 | 115843445 | 0.4306 | 0.0664 | 8.89E-11 | 700748 | A | G | 0.0578 | 42.05444 |
| ADRB1 | rs11196553 | 10 | 115710997 | 0.6252 | 0.074 | 2.89E-17 | 720063 | T | C | 0.045 | 71.37966 |
| ADRB1 | rs460718 | 10 | 115721364 | 0.2764 | 0.0324 | 1.36E-17 | 725026 | G | A | 0.6734 | 72.77564 |
| ADRB1 | rs79850079 | 10 | 115790006 | -0.5804 | 0.0905 | 1.45E-10 | 693367 | A | G | 0.0317 | 41.1299 |
| ADRB1 | rs17875473 | 10 | 115800294 | 0.3283 | 0.0552 | 2.66E-09 | 707302 | T | C | 0.0871 | 35.37233 |
| ADRB1 | rs68122733 | 10 | 115831533 | -0.3343 | 0.0406 | 1.69E-16 | 715593 | G | A | 0.1718 | 67.79859 |
| ADRB1 | rs180898 | 10 | 115765397 | -0.5177 | 0.065 | 1.65E-15 | 649501 | C | A | 0.0661 | 63.4351 |
| ADRB1 | rs11196597 | 10 | 115788094 | 0.2858 | 0.0458 | 4.23E-10 | 701272 | A | G | 0.133 | 38.93978 |
| ADRB1 | rs11196625 | 10 | 115843990 | 0.2274 | 0.0395 | 8.29E-09 | 698363 | A | G | 0.1928 | 33.14261 |
| CACNA1C | rs2239046 | 12 | 2434419 | 0.2082 | 0.0322 | 9.58E-11 | 739131 | A | G | 0.6817 | 41.80707 |
| CACNA1C | rs714277 | 12 | 2514270 | 0.1986 | 0.0333 | 2.38E-09 | 738689 | T | C | 0.2834 | 35.56887 |
| CACNA1D | rs113210396 | 3 | 53612327 | -0.4338 | 0.077 | 1.76E-08 | 664156 | T | G | 0.0451 | 31.73932 |
| CACNA1D | rs3821843 | 3 | 53558012 | 0.3373 | 0.0335 | 6.56E-24 | 684820 | A | G | 0.6808 | 101.3778 |
| CACNA1D | rs9311502 | 3 | 53560321 | 0.2463 | 0.0355 | 3.87E-12 | 727145 | C | T | 0.2391 | 48.13623 |
| CACNA1D | rs62250937 | 3 | 53870318 | -0.3238 | 0.056 | 7.28E-09 | 655229 | C | T | 0.0893 | 33.43318 |
| CACNA1D | rs114718455 | 3 | 53464055 | 0.5102 | 0.0905 | 1.72E-08 | 640928 | G | A | 0.0336 | 31.78218 |
| CACNA1D | rs114987861 | 3 | 53605712 | 0.5289 | 0.0958 | 3.36E-08 | 680765 | A | G | 0.0284 | 30.48008 |
| CACNA1D | rs2633731 | 3 | 53738424 | 0.1963 | 0.0309 | 2.21E-10 | 725982 | C | T | 0.6038 | 40.35744 |
| CACNA1D | rs312487 | 3 | 53545622 | -0.2194 | 0.0307 | 9.65E-13 | 706661 | C | T | 0.5217 | 51.0736 |
| CACNA1D | rs7340705 | 3 | 53734443 | 0.2425 | 0.0322 | 4.87E-14 | 727632 | C | T | 0.3268 | 56.7168 |
| CACNA1D | rs3774472 | 3 | 53638200 | 0.1747 | 0.0303 | 8.21E-09 | 723931 | G | A | 0.4943 | 33.24303 |
| CCNB2 | rs1757213 | 10 | 18537594 | -0.3084 | 0.0507 | 1.15E-09 | 669049 | G | A | 0.888 | 37.00095 |
| CCNB2 | rs7077127 | 10 | 18717348 | 0.217 | 0.0334 | 7.95E-11 | 730909 | T | G | 0.2864 | 42.21109 |
| CCNB2 | rs72786098 | 10 | 18729855 | -0.5033 | 0.0883 | 1.18E-08 | 705121 | A | G | 0.0322 | 32.48871 |
| CCNB2 | rs17604757 | 10 | 18442940 | 0.5022 | 0.0606 | 1.12E-16 | 725278 | G | A | 0.0675 | 68.6765 |
| CCNB2 | rs17610275 | 10 | 18621630 | -0.3868 | 0.0613 | 2.87E-10 | 669382 | G | T | 0.0734 | 39.81548 |
| CCNB2 | rs11014021 | 10 | 18682233 | -0.3245 | 0.0307 | 4.29E-26 | 724968 | A | T | 0.4182 | 111.7256 |
| CCNB2 | rs7923191 | 10 | 18727901 | 0.369 | 0.0376 | 1.09E-22 | 713691 | G | A | 0.2082 | 96.31126 |
| CCNB2 | rs10828749 | 10 | 18756881 | -0.3658 | 0.0309 | 2.27E-32 | 718094 | A | G | 0.412 | 140.1427 |
| CCNB2 | rs4748472 | 10 | 18776197 | 0.3161 | 0.0319 | 4.04E-23 | 725324 | T | C | 0.6558 | 98.19008 |
| CCNB2 | rs12416030 | 10 | 18789075 | 0.2088 | 0.0381 | 4.32E-08 | 710150 | C | T | 0.2031 | 30.03385 |
| CCNB2 | rs75699707 | 10 | 18359294 | -0.5761 | 0.1028 | 2.10E-08 | 640524 | A | G | 0.0264 | 31.40578 |
| CCNB2 | rs10764319 | 10 | 18428415 | 0.2693 | 0.0329 | 2.54E-16 | 728693 | T | C | 0.3044 | 67.00094 |
| CCNB2 | rs12765240 | 10 | 18438372 | 0.396 | 0.052 | 2.52E-14 | 730898 | T | C | 0.0932 | 57.99408 |
| CCNB2 | rs11012811 | 10 | 18438456 | 0.3095 | 0.0326 | 2.31E-21 | 730317 | T | G | 0.31 | 90.13347 |
| CCNB2 | rs4748444 | 10 | 18494482 | -0.1939 | 0.0327 | 3.13E-09 | 700079 | C | T | 0.3363 | 35.16091 |
| CCNB2 | rs982003 | 10 | 18707296 | -0.2414 | 0.0351 | 6.21E-12 | 734615 | T | C | 0.7568 | 47.29991 |
| CCNB2 | rs7076247 | 10 | 18759629 | -0.2557 | 0.0309 | 1.33E-16 | 736767 | C | T | 0.6114 | 68.47696 |
| CCNB2 | rs1888693 | 10 | 18440444 | 0.3858 | 0.0317 | 4.69E-34 | 732344 | A | G | 0.3449 | 148.1173 |
| CCNB2 | rs61278674 | 10 | 18481737 | 0.3298 | 0.054 | 1.03E-09 | 676191 | G | A | 0.0938 | 37.30043 |
| CCNB2 | rs10828399 | 10 | 18553968 | -0.1947 | 0.0302 | 1.10E-10 | 732842 | A | G | 0.5218 | 41.56407 |
| CCNB2 | rs11013910 | 10 | 18663370 | 0.2672 | 0.0449 | 2.70E-09 | 728662 | A | G | 0.1306 | 35.41443 |
| CCNB2 | rs11013938 | 10 | 18669271 | -0.3265 | 0.035 | 1.17E-20 | 715103 | C | G | 0.2554 | 87.02224 |
| CCNB2 | rs12258967 | 10 | 18727959 | -0.6327 | 0.0337 | 1.08E-78 | 711222 | G | C | 0.2953 | 352.4811 |
| CCNB2 | rs7917532 | 10 | 18373902 | -0.2323 | 0.0304 | 2.01E-14 | 723522 | C | T | 0.5347 | 58.39172 |
| CCNB2 | rs7074171 | 10 | 18676897 | 0.3061 | 0.0314 | 1.76E-22 | 695584 | G | A | 0.5795 | 95.03145 |
| CCNB2 | rs67214975 | 10 | 18727251 | -0.4144 | 0.0307 | 1.42E-41 | 714644 | A | C | 0.4563 | 182.206 |
| CCNB2 | rs10828784 | 10 | 18788273 | -0.2021 | 0.0345 | 4.49E-09 | 633311 | G | C | 0.3367 | 34.31582 |
| CCNB2 | rs12416052 | 10 | 18789267 | -0.1987 | 0.0311 | 1.59E-10 | 717434 | C | T | 0.4053 | 40.82018 |
| CCNB2 | rs74593582 | 10 | 18374059 | 0.7581 | 0.1349 | 1.91E-08 | 616746 | C | T | 0.0154 | 31.58123 |
| CCNB2 | rs6482184 | 10 | 18403118 | 0.1949 | 0.0316 | 6.65E-10 | 712976 | C | T | 0.3795 | 38.04079 |
| CCNB2 | rs16916922 | 10 | 18467744 | 0.3662 | 0.0433 | 2.86E-17 | 731281 | T | A | 0.1415 | 71.5255 |
| CCNB2 | rs1757225 | 10 | 18516925 | -0.2453 | 0.0325 | 4.29E-14 | 730147 | G | A | 0.6855 | 56.96766 |
| CCNB2 | rs10828452 | 10 | 18592450 | -0.3046 | 0.0388 | 4.20E-15 | 679250 | T | A | 0.207 | 61.63059 |
| CCNB2 | rs10828542 | 10 | 18627285 | -0.1817 | 0.0311 | 5.18E-09 | 725325 | G | A | 0.3863 | 34.13415 |
| CCNB2 | rs112701401 | 10 | 18644811 | -0.5026 | 0.0921 | 4.91E-08 | 668265 | G | C | 0.0305 | 29.78007 |
| CCNB2 | rs112133583 | 10 | 18695681 | -0.5546 | 0.0973 | 1.18E-08 | 625088 | T | C | 0.0299 | 32.48883 |
| CCNB2 | rs72786085 | 10 | 18713206 | -0.5309 | 0.0595 | 4.46E-19 | 662090 | C | G | 0.0792 | 79.61438 |
| CCNB2 | rs116936375 | 10 | 18737135 | -0.5739 | 0.081 | 1.40E-12 | 673629 | A | G | 0.0405 | 50.19985 |
| CCNB2 | rs7100884 | 10 | 18812697 | 0.2064 | 0.0367 | 1.85E-08 | 731989 | G | A | 0.7845 | 31.62913 |
| CCNB2 | rs12778700 | 10 | 18385490 | -0.1972 | 0.033 | 2.24E-09 | 694041 | C | T | 0.3323 | 35.70968 |
| CCNB2 | rs1539680 | 10 | 18502889 | -0.3259 | 0.0375 | 3.37E-18 | 722972 | C | G | 0.7929 | 75.52769 |
| CCNB2 | rs1891392 | 10 | 18336421 | -0.2276 | 0.0334 | 9.02E-12 | 731414 | C | T | 0.7129 | 46.43566 |
| CCNB2 | rs1998822 | 10 | 18755664 | 0.1958 | 0.0343 | 1.15E-08 | 707468 | G | A | 0.2766 | 32.58646 |
| CCNB3 | rs150857355 | 12 | 49209340 | 0.9406 | 0.1122 | 5.20E-17 | 650268 | C | G | 0.0217 | 70.27878 |
| CCNB3 | rs17123362 | 12 | 49255964 | 0.4067 | 0.0672 | 1.41E-09 | 686446 | A | G | 0.0586 | 36.62771 |

SNP, single-nucleotide polymorphism; chr, chromosome; beta: effect size; se, standard errors; eaf, effect allele frequency; pos, position; F, F-statistic = (beta/se)^2^.

**Table S3. Results of Mendelian randomization analysis related to antihypertensive drugs and positive controls.**

| **Outcome** | **Drug** | **Method** | **NSNP** | **Beta** | **SE** | **P Value** | **Lo_ci** | **Up_ci** | **OR** | **OR_lci95** | **OR_uci95** |
| --- | --- | --- | --- | --- | --- | --- | --- | --- | --- | --- | --- |
| Hypertension | ACEIs | Wald ratio | 1 | -0.12648 | 0.03188 | 0.00007 | -0.18896 | -0.06401 | 0.88119 | 0.82782 | 0.93800 |
| Hypertension | BBs | Inverse variance weighted (multiplicative random effects) | 12 | -0.15225 | 0.01489 | 0.00000 | -0.18144 | -0.12306 | 0.85878 | 0.83407 | 0.88421 |
|  |  | MR Egger | 12 | -0.15443 | 0.05842 | 0.02459 | -0.26894 | -0.03993 | 0.85690 | 0.76419 | 0.96086 |
|  |  | Weighted median | 12 | -0.16124 | 0.01730 | 0.00000 | -0.19514 | -0.12734 | 0.85109 | 0.82272 | 0.88044 |
|  |  | Simple mode | 12 | -0.18662 | 0.02825 | 0.00004 | -0.24198 | -0.13126 | 0.82976 | 0.78507 | 0.87699 |
|  |  | Weighted mode | 12 | -0.18427 | 0.02420 | 0.00001 | -0.23169 | -0.13684 | 0.83171 | 0.79319 | 0.87211 |
| Hypertension | CCBs | Inverse variance weighted | 56 | -0.09337 | 0.00738 | 0.00000 | -0.10783 | -0.07890 | 0.91086 | 0.89778 | 0.92413 |
|  |  | Inverse variance weighted | 56 | -0.09337 | 0.00738 | 0.00000 | -0.10783 | -0.07890 | 0.91086 | 0.89778 | 0.92413 |
|  |  | MR Egger | 56 | -0.11255 | 0.02118 | 0.00000 | -0.15406 | -0.07104 | 0.89355 | 0.85722 | 0.93143 |
|  |  | Weighted median | 56 | -0.10000 | 0.00896 | 0.00000 | -0.11756 | -0.08244 | 0.90484 | 0.88909 | 0.92087 |
|  |  | Simple mode | 56 | -0.09953 | 0.01885 | 0.00000 | -0.13648 | -0.06258 | 0.90526 | 0.87243 | 0.93933 |
|  |  | Weighted mode | 56 | -0.10234 | 0.01362 | 0.00000 | -0.12904 | -0.07564 | 0.90272 | 0.87894 | 0.92715 |
| Coronary atherosclerosis | ACEIs | Wald ratio | 1 | -0.08986 | 0.043744 | 0.03995 | -0.1756 | -0.00412 | 0.914058 | 0.838955 | 0.995885 |
| Coronary atherosclerosis | BBs | Inverse variance weighted | 12 | -0.04596 | 0.01474 | 0.00182 | -0.07486 | -0.01707 | 0.95508 | 0.92787 | 0.98308 |
|  |  | MR Egger | 12 | -0.02913 | 0.05669 | 0.61847 | -0.14025 | 0.08198 | 0.97129 | 0.86914 | 1.08543 |
|  |  | Weighted median | 12 | -0.05080 | 0.01985 | 0.01048 | -0.08970 | -0.01190 | 0.95047 | 0.91420 | 0.98817 |
|  |  | Simple mode | 12 | -0.04895 | 0.02890 | 0.11846 | -0.10560 | 0.00770 | 0.95223 | 0.89979 | 1.00773 |
|  |  | Weighted mode | 12 | -0.05203 | 0.02455 | 0.05762 | -0.10015 | -0.00391 | 0.94930 | 0.90470 | 0.99609 |
| Coronary atherosclerosis | CCBs | Inverse variance weighted | 56 | -0.01714 | 0.00712 | 0.01612 | -0.03110 | -0.00318 | 0.98301 | 0.96938 | 0.99683 |
|  |  | MR Egger | 56 | -0.04113 | 0.02042 | 0.04897 | -0.08115 | -0.00111 | 0.95971 | 0.92206 | 0.99889 |
|  |  | Weighted median | 56 | -0.02801 | 0.01045 | 0.00735 | -0.04849 | -0.00753 | 0.97238 | 0.95267 | 0.99250 |
|  |  | Simple mode | 56 | -0.02224 | 0.02226 | 0.32211 | -0.06586 | 0.02139 | 0.97801 | 0.93626 | 1.02162 |
|  |  | Weighted mode | 56 | -0.03224 | 0.01690 | 0.06170 | -0.06538 | 0.00089 | 0.96827 | 0.93671 | 1.00089 |

Beta, standardized causal point estimate; Lo_ci, lower bound for 95% confidence interval based on standard error; Up_ci, upper bound for 95% confidence interval based on standard error; NSNP, number of instrument variants used in the calculation; OR, odds ratio; P Value, p-value associated with causal point estimate; SE, standard error associated with causal point estimate; SNP, single nucleotide polymorphisms.

**Table S4. Results of heterogeneity and pleiotropy tests related to antihypertensive drugs and positive controls.**

| **Outcome** | **Drug** | **Method** | **NSNP** | **Q**  **(MR Egger)** | **Q_df (MR Egger)** | **Q_pval (MR Egger)** | **Q(IVW)** | **Q_df (IVW)** | **Q_pval (IVW)** | **Egger_intercept** | **Se (egger_intercept)** | **Pval (egger_intercept)** |
| --- | --- | --- | --- | --- | --- | --- | --- | --- | --- | --- | --- | --- |
| Hypertension | ACEIs | Wald ratio | 1 | NA | NA | NA | NA | NA | NA | NA | NA | NA |
| Hypertension | BBs | Inverse variance weighted (multiplicative random effects) | 12 | 21.1655 | 10 | 0.019969 | 21.16869 | 11 | 0.031665 | 0.0008222 | 0.02115168 | 0.96975769 |
| Hypertension | CCBs | Inverse variance weighted (multiplicative random effects) | 56 | 109.8839 | 54 | 1.09E-05 | 111.7843 | 55 | 9.53E-06 | 0.00614845 | 0.0063624 | 0.33816576 |
| Coronary atherosclerosis | ACEIs | Wald ratio | 1 | NA | NA | NA | NA | NA | NA | NA | NA | NA |
| Coronary atherosclerosis | BBs | Inverse variance weighted | 12 | 10.57385 | 10 | 0.391668 | 10.6742 | 11 | 0.470947 | -0.0063256 | 0.02053247 | 0.76434429 |
| Coronary atherosclerosis | CCBs | Inverse variance weighted | 56 | 52.47081 | 54 | 0.533551 | 54.04245 | 55 | 0.511218 | 0.00768695 | 0.00613165 | 0.21536908 |

NSNP, number of instrument variants used in the calculation; Q_pval, p-value of Cochran’s Q statistic; Q, heterogeneity statistic (Cochran’s Q statistic); Q_df, degrees of freedom for Q statistic.

**Table S5. Results of Mendelian randomization analysis related to antihypertensive drugs and COVID-19 outcomes.**

| **Outcome** | **Drug** | **Method** | **NSNP** | **Beta** | **SE** | **P Value** | **Lo_ci** | **Up_ci** | **OR** | **OR_lci95** | **OR_uci95** |
| --- | --- | --- | --- | --- | --- | --- | --- | --- | --- | --- | --- |
| COVID-19 susceptibility | ACEIs | Wald ratio | 1 | 0.007298 | 0.016533 | 0.658892 | -0.02511 | 0.039703 | 1.007325 | 0.975206 | 1.040502 |
| COVID-19 susceptibility | BBs | Inverse variance weighted | 12 | -0.00107 | 0.005423 | 0.843068 | -0.0117 | 0.009556 | 0.998927 | 0.988365 | 1.009602 |
|  |  | MR Egger | 12 | 0.00957 | 0.02058 | 0.65178 | -0.03077 | 0.04992 | 1.00962 | 0.96970 | 1.05118 |
|  |  | Weighted median | 12 | -0.00008 | 0.00758 | 0.99118 | -0.01495 | 0.01478 | 0.99992 | 0.98516 | 1.01489 |
|  |  | Simple mode | 12 | -0.01079 | 0.01192 | 0.38478 | -0.03415 | 0.01257 | 0.98927 | 0.96643 | 1.01265 |
|  |  | Weighted mode | 12 | 0.00227 | 0.01008 | 0.82592 | -0.01748 | 0.02202 | 1.00227 | 0.98267 | 1.02227 |
| COVID-19 susceptibility | CCBs | Inverse variance weighted | 57 | -0.00656 | 0.002607 | 0.011791 | -0.01167 | -0.00146 | 0.993457 | 0.988394 | 0.998546 |
|  |  | MR Egger | 57 | -0.00282 | 0.00750 | 0.70860 | -0.01752 | 0.01188 | 0.99719 | 0.98263 | 1.01195 |
|  |  | Weighted median | 57 | -0.00788 | 0.00390 | 0.04333 | -0.01551 | -0.00024 | 0.99216 | 0.98460 | 0.99976 |
|  |  | Simple mode | 57 | -0.00768 | 0.00679 | 0.26305 | -0.02100 | 0.00563 | 0.99235 | 0.97922 | 1.00565 |
|  |  | Weighted mode | 57 | -0.00799 | 0.00518 | 0.12815 | -0.01814 | 0.00215 | 0.99204 | 0.98203 | 1.00215 |
| COVID-19 hospitalization | ACEIs | Wald ratio | 1 | 0.018779 | 0.03607 | 0.602634 | -0.05192 | 0.089475 | 1.018956 | 0.949406 | 1.093601 |
| COVID-19 hospitalization | BBs | Inverse variance weighted | 12 | 0.003482 | 0.011304 | 0.758053 | -0.01867 | 0.025637 | 1.003488 | 0.9815 | 1.025968 |
|  |  | MR Egger | 12 | 0.06049 | 0.04210 | 0.18135 | -0.02203 | 0.14301 | 1.06235 | 0.97821 | 1.15374 |
|  |  | Weighted median | 12 | 0.00245 | 0.01502 | 0.87049 | -0.02700 | 0.03190 | 1.00245 | 0.97336 | 1.03241 |
|  |  | Simple mode | 12 | 0.00049 | 0.02322 | 0.98342 | -0.04501 | 0.04600 | 1.00049 | 0.95598 | 1.04708 |
|  |  | Weighted mode | 12 | -0.00061 | 0.02059 | 0.97683 | -0.04096 | 0.03974 | 0.99939 | 0.95986 | 1.04054 |
| COVID-19 hospitalization | CCBs | Inverse variance weighted | 57 | -0.00639 | 0.005918 | 0.280373 | -0.01799 | 0.005211 | 0.993632 | 0.982173 | 1.005225 |
|  |  | MR Egger | 57 | 0.02055 | 0.01670 | 0.22365 | -0.01218 | 0.05328 | 1.02076 | 0.98790 | 1.05473 |
|  |  | Weighted median | 57 | -0.00017 | 0.00846 | 0.98424 | -0.01675 | 0.01641 | 0.99983 | 0.98339 | 1.01655 |
|  |  | Simple mode | 57 | -0.01289 | 0.01693 | 0.44942 | -0.04607 | 0.02028 | 0.98719 | 0.95498 | 1.02049 |
|  |  | Weighted mode | 57 | -0.00218 | 0.01252 | 0.86238 | -0.02673 | 0.02237 | 0.99782 | 0.97363 | 1.02262 |
| COVID-19 severe disease | ACEIs | Wald ratio | 1 | 0.048745 | 0.052024 | 0.348774 | -0.05322 | 0.150713 | 1.049953 | 0.948169 | 1.162663 |
| COVID-19 severe disease | BBs | Inverse variance weighted | 12 | 0.003861 | 0.020935 | 0.853692 | -0.03717 | 0.044893 | 1.003868 | 0.963511 | 1.045916 |
|  |  | MR Egger | 12 | 0.03479 | 0.08243 | 0.68188 | -0.12677 | 0.19636 | 1.03541 | 0.88094 | 1.21696 |
|  |  | Weighted median | 12 | 0.00818 | 0.02280 | 0.71989 | -0.03651 | 0.05286 | 1.00821 | 0.96415 | 1.05429 |
|  |  | Simple mode | 12 | 0.00602 | 0.03073 | 0.84816 | -0.05421 | 0.06625 | 1.00604 | 0.94724 | 1.06850 |
|  |  | Weighted mode | 12 | 0.00602 | 0.02442 | 0.80972 | -0.04185 | 0.05389 | 1.00604 | 0.95902 | 1.05537 |
| COVID-19 severe disease | CCBs | Inverse variance weighted (multiplicative random effects) | 57 | 0.00044 | 0.009663 | 0.963651 | -0.0185 | 0.019381 | 1.00044 | 0.98167 | 1.01957 |
|  |  | MR Egger | 57 | 0.05365 | 0.02762 | 0.05720 | -0.00048 | 0.10778 | 1.05511 | 0.99952 | 1.11380 |
|  |  | Weighted median | 57 | 0.00506 | 0.01288 | 0.69424 | -0.02018 | 0.03030 | 1.00507 | 0.98003 | 1.03076 |
|  |  | Simple mode | 57 | 0.02959 | 0.02804 | 0.29579 | -0.02536 | 0.08454 | 1.03003 | 0.97496 | 1.08822 |
|  |  | Weighted mode | 57 | 0.01802 | 0.01968 | 0.36369 | -0.02054 | 0.05658 | 1.01818 | 0.97967 | 1.05821 |

Beta, standardized causal point estimate; Lo_ci, lower bound for 95% confidence interval based on standard error; Up_ci, upper bound for 95% confidence interval based on standard error; NSNP, number of instrument variants used in the calculation; OR, odds ratio; P Value, p-value associated with causal point estimate; SE, standard error associated with causal point estimate; SNP, single nucleotide polymorphisms.

**Table S6. Results of heterogeneity and pleiotropy tests related to antihypertensive drugs and COVID-19 outcomes.**

| **Outcome** | **Drug** | **Method** | **Q(MR Egger)** | **Q_df(MR Egger)** | **Q_pval(MR Egger)** | **Q(IVW)** | **Q_df(IVW)** | **Q_pval(IVW)** | **egger_intercept** | **se(egger_intercept)** | **pval(egger_intercept)** |
| --- | --- | --- | --- | --- | --- | --- | --- | --- | --- | --- | --- |
| COVID-19 susceptibility | ACEIs | Wald ratio | NA | NA | NA | NA | NA | NA | NA | NA | NA |
| COVID-19 susceptibility | BBs | Inverse variance weighted | 11.17179 | 10 | 0.344292 | 11.49474 | 11 | 0.402793 | -0.00407 | 0.007564 | 0.602573 |
| COVID-19 susceptibility | CCBs | Inverse variance weighted | 57.70172 | 55 | 0.375675 | 58.00006 | 56 | 0.40143 | -0.00121 | 0.002276 | 0.595997 |
| COVID-19 hospitalization | ACEIs | Wald ratio | NA | NA | NA | NA | NA | NA | NA | NA | NA |
| COVID-19 hospitalization | BBs | Inverse variance weighted | 5.712664 | 10 | 0.838799 | 7.688364 | 11 | 0.740918 | -0.02197 | 0.015632 | 0.190144 |
| COVID-19 hospitalization | CCBs | Inverse variance weighted | 60.21454 | 55 | 0.292731 | 63.45775 | 56 | 0.230253 | -0.00871 | 0.005061 | 0.090846 |
| COVID-19 severe disease | ACEIs | Wald ratio | NA | NA | NA | NA | NA | NA | NA | NA | NA |
| COVID-19 severe disease | BBs | Inverse variance weighted | 17.04773 | 10 | 0.073314 | 17.30586 | 11 | 0.099148 | -0.012 | 0.030844 | 0.705344 |
| COVID-19 severe disease | CCBs | Inverse variance weighted (multiplicative random effects) | 69.80786 | 55 | 0.086241 | 75.13587 | 56 | 0.044855 | -0.01695 | 0.008271 | 0.045259 |

NSNP, number of instrument variants used in the calculation; Q_pval, p-value of Cochran’s Q statistic; Q, heterogeneity statistic (Cochran’s Q statistic); Q_df, degrees of freedom for Q statistic.

**Table S7. Mendelian randomization analysis of systolic pressure on COVID-19 susceptibility** **(other than CCB targets).**

| **Exposure** | **Outcome** | **Method** | | **NSNP** | **Beta** | **SE** | **P Value** | **Lo_ci** | **Up_ci** | **OR** | **OR_lci95** | **OR_uci95** |
| --- | --- | --- | --- | --- | --- | --- | --- | --- | --- | --- | --- | --- |
| Systolic blood pressure | COVID-19 susceptibility | Inverse variance weighted (multiplicative random effects) | 311 | | -0.00079 | 0.001164 | 0.494813 | -0.00308 | 0.001487 | 0.999205 | 0.996928 | 1.001488 |

Beta, standardized causal point estimate; Lo_ci, lower bound for 95% confidence interval based on standard error; Up_ci, upper bound for 95% confidence interval based on standard error; NSNP, number of instrument variants used in the calculation; OR, odds ratio; P Value, p-value associated with causal point estimate; SE, standard error associated with causal point estimate; SNP, single nucleotide polymorphisms. Clump parameter: r2=0.001, p1 = 5e-08, kb=10000.

**Table S8. Results of Mendelian randomization analyses validating the association of antihypertensive drugs and COVID-19 outcomes in the diastolic blood pressure cohort**

| **Outcome** | **Drug** | **Method** | **NSNP** | **Beta** | **SE** | **P Value** | **Lo_ci** | **Up_ci** | **OR** | **OR_lci95** | **OR_uci95** |
| --- | --- | --- | --- | --- | --- | --- | --- | --- | --- | --- | --- |
| COVID-19 susceptibility | ACEIs | Wald ratio | 1 | 0.01195 | 0.02707 | 0.65889 | -0.04111 | 0.06501 | 1.01202 | 0.95972 | 1.06717 |
| COVID-19 susceptibility | BBs | Inverse variance weighted | 12 | -0.00177 | 0.00801 | 0.82489 | -0.01746 | 0.01392 | 0.99823 | 0.98269 | 1.01402 |
|  |  | MR Egger | 12 | 0.01324 | 0.03228 | 0.69045 | -0.05004 | 0.07651 | 1.01332 | 0.95120 | 1.07951 |
|  |  | Weighted median | 12 | -0.00058 | 0.01113 | 0.95875 | -0.02240 | 0.02125 | 0.99942 | 0.97785 | 1.02147 |
|  |  | Simple mode | 12 | -0.01576 | 0.01941 | 0.43404 | -0.05381 | 0.02228 | 0.98436 | 0.94762 | 1.02253 |
|  |  | Weighted mode | 12 | 0.00098 | 0.01545 | 0.95039 | -0.02929 | 0.03126 | 1.00098 | 0.97113 | 1.03175 |
| COVID-19 susceptibility | CCBs | Inverse variance weighted | 57 | -0.01217 | 0.00457 | 0.00775 | -0.02113 | -0.00321 | 0.98790 | 0.97909 | 0.99679 |
|  |  | MR Egger | 57 | -0.00963 | 0.01286 | 0.45728 | -0.03483 | 0.01558 | 0.99042 | 0.96577 | 1.01570 |
|  |  | Weighted median | 57 | -0.01378 | 0.00642 | 0.03186 | -0.02637 | -0.00120 | 0.98631 | 0.97398 | 0.99881 |
|  |  | Simple mode | 57 | -0.01478 | 0.01148 | 0.20313 | -0.03728 | 0.00772 | 0.98533 | 0.96341 | 1.00775 |
|  |  | Weighted mode | 57 | -0.01430 | 0.00889 | 0.11313 | -0.03172 | 0.00312 | 0.98580 | 0.96877 | 1.00312 |
| COVID-19 hospitalization | ACEIs | Wald ratio | 1 | 0.03075 | 0.05906 | 0.60263 | -0.08501 | 0.14651 | 1.03123 | 0.91850 | 1.15779 |
| COVID-19 hospitalization | BBs | Inverse variance weighted | 12 | 0.00394 | 0.01672 | 0.81385 | -0.02884 | 0.03671 | 1.00395 | 0.97157 | 1.03740 |
|  |  | MR Egger | 12 | 0.07982 | 0.06526 | 0.24932 | -0.04809 | 0.20773 | 1.08309 | 0.95305 | 1.23088 |
|  |  | Weighted median | 12 | 0.00364 | 0.02271 | 0.87265 | -0.04086 | 0.04814 | 1.00365 | 0.95996 | 1.04932 |
|  |  | Simple mode | 12 | 0.00070 | 0.03503 | 0.98451 | -0.06797 | 0.06936 | 1.00070 | 0.93429 | 1.07182 |
|  |  | Weighted mode | 12 | -0.00159 | 0.02704 | 0.95430 | -0.05459 | 0.05142 | 0.99842 | 0.94687 | 1.05277 |
| COVID-19 hospitalization | CCBs | Inverse variance weighted | 57 | -0.01055 | 0.01046 | 0.31307 | -0.03104 | 0.00995 | 0.98951 | 0.96943 | 1.01000 |
|  |  | MR Egger | 57 | 0.04044 | 0.02883 | 0.16630 | -0.01606 | 0.09695 | 1.04127 | 0.98406 | 1.10180 |
|  |  | Weighted median | 57 | 0.00131 | 0.01507 | 0.93064 | -0.02822 | 0.03084 | 1.00131 | 0.97217 | 1.03133 |
|  |  | Simple mode | 57 | -0.01388 | 0.02913 | 0.63549 | -0.07098 | 0.04321 | 0.98621 | 0.93148 | 1.04416 |
|  |  | Weighted mode | 57 | 0.00108 | 0.02155 | 0.96005 | -0.04115 | 0.04332 | 1.00108 | 0.95968 | 1.04427 |
| COVID-19 severe disease | ACEIs | Wald ratio | 1 | 0.07982 | 0.08519 | 0.34877 | -0.08715 | 0.24678 | 1.08309 | 0.91654 | 1.27990 |
| COVID-19 severe disease | BBs | Inverse variance weighted | 12 | 0.00574 | 0.03102 | 0.85325 | -0.05505 | 0.06653 | 1.00575 | 0.94643 | 1.06879 |
|  |  | MR Egger | 12 | 0.05590 | 0.12687 | 0.66888 | -0.19277 | 0.30457 | 1.05749 | 0.82467 | 1.35604 |
|  |  | Weighted median | 12 | 0.01201 | 0.03373 | 0.72177 | -0.05410 | 0.07813 | 1.01208 | 0.94734 | 1.08126 |
|  |  | Simple mode | 12 | 0.00734 | 0.04337 | 0.86863 | -0.07767 | 0.09236 | 1.00737 | 0.92527 | 1.09675 |
|  |  | Weighted mode | 12 | 0.00734 | 0.03579 | 0.84116 | -0.06280 | 0.07748 | 1.00737 | 0.93914 | 1.08056 |
| COVID-19 severe disease | CCBs | Inverse variance weighted | 57 | 0.00127 | 0.01703 | 0.94059 | -0.03211 | 0.03465 | 1.00127 | 0.96840 | 1.03525 |
|  |  | MR Egger | 57 | 0.09692 | 0.04811 | 0.04886 | 0.00262 | 0.19123 | 1.10178 | 1.00262 | 1.21073 |
|  |  | Weighted median | 57 | 0.01522 | 0.02253 | 0.49941 | -0.02894 | 0.05937 | 1.01533 | 0.97147 | 1.06117 |
|  |  | Simple mode | 57 | 0.04610 | 0.05000 | 0.36050 | -0.05190 | 0.14410 | 1.04718 | 0.94942 | 1.15500 |
|  |  | Weighted mode | 57 | 0.03209 | 0.03429 | 0.35328 | -0.03511 | 0.09930 | 1.03261 | 0.96550 | 1.10439 |

Beta, standardized causal point estimate; Lo_ci, lower bound for 95% confidence interval based on standard error; Up_ci, upper bound for 95% confidence interval based on standard error; NSNP, number of instrument variants used in the calculation; OR, odds ratio; P Value, p-value associated with causal point estimate; SE, standard error associated with causal point estimate; SNP, single nucleotide polymorphisms

**Figure S1. Sensitivity analysis of the results of mendelian randomization.**

**
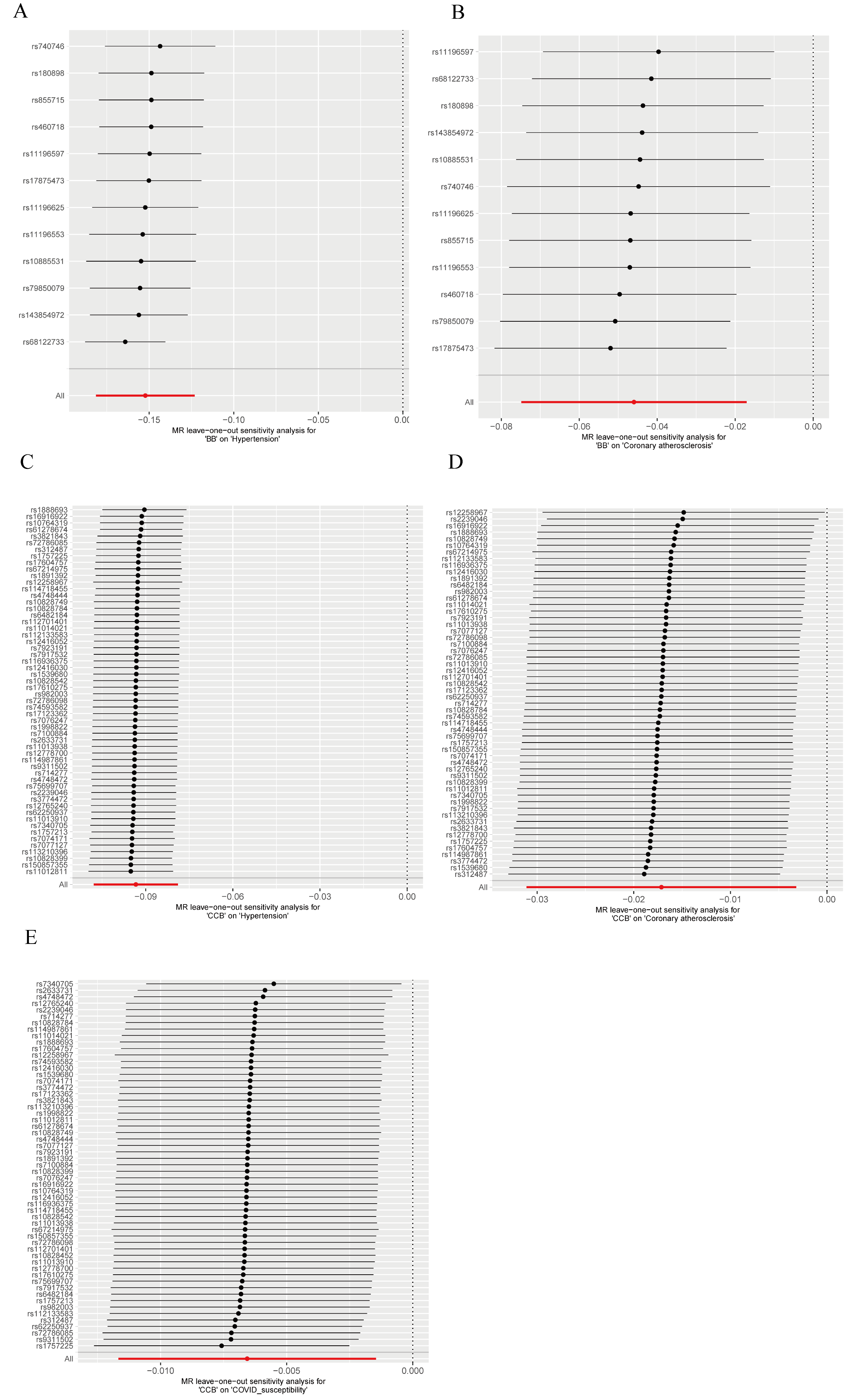
**

A. MR leave−one−out sensitivity analysis for'BB' on 'Hypertension'. B. MR leave−one−out sensitivity analysis for'BB' on ' Coronary atherosclerosis'. C. MR leave−one−out sensitivity analysis for'CCB' on 'Hypertension'. D. MR leave−one−out sensitivity analysis for'CCB' on ' Coronary atherosclerosis'. E. MR leave−one−out sensitivity analysis for 'CCB' on' COVID-19 susceptibility'. B: β-blocker; CCB: calcium channel blockers; MR: Mendelian randomization.
